# Stereotaktisch ablative Strahlentherapie für operable nichtkleinzellige Bronchialkarzinome in Stadium I (überarbeitete STARS-Studie): Langzeitergebnisse einer einarmigen, prospektiven Studie mit Vergleich zur Operation

**DOI:** 10.1007/s00066-021-01893-z

**Published:** 2021-12-20

**Authors:** Kim Melanie Kraus, Stephanie Elisabeth Combs

**Affiliations:** 1 Klinik für Strahlentherapie und RadioOnkologie des Klinikums rechts der Isar, Technische Universität München (TUM), Ismaninger Straße 22, 81675 München, Deutschland; 2 Institute of Radiation Medicine (IRM), Helmholtz Zentrum München, Neuherberg, Deutschland; 3 Partner Site Munich, Deutsches Konsortium für Translationale Krebsforschung (DKTK), München, Deutschland

## Ziel und Hintergrund

Die gepoolte Analyse der STARS- und ROSEL-Studien lieferte sehr positive Ergebnisse bezüglich des Therapieerfolgs und des Überlebens nach einer stereotaktischen Strahlentherapie (engl. SBRT für „stereotactic body radiation therapy“). Allerdings hatten diese Studien auch deutliche Limitationen, insbesondere die mangelhafte Rekrutierung stellt ein Problem bei der Bewertung der Daten dar. Bislang fehlen Langzeitdaten aus großen randomisierten Studien. Deshalb haben die Autoren der hier kommentierten Arbeit einen Vergleich mit der Propensity-score-matching-Methode angestellt zwischen einem größeren Datensatz der überarbeiteten STARS-Studie und einem prospektiv gesammelten Datensatz von Patienten, welche eine videoassistierte thorakoskopische Lobektomie inklusive mediastinaler Lymphknotendissektion (VATS L-MLND) erhalten hatten.

## Methode/Patientengut

Es wurden Patienten mit einem Mindestalter von 18 Jahren und einem Zubrod-Index zwischen 0 und 2 eingeschlossen. Alle Patienten litten unter einem neu diagnostizierten, histologisch bestätigten nichtkleinzelligen Bronchialkarzinom (NSCLC, engl. für „non-small-cell lung cancer“) ohne Lymphknotenbefall (N0) oder Fernmetastasierung (M0) von maximal 3 cm Durchmesser. Alle Patienten erhielten eine ^18^F‑Fluordesoxyglukose-Positronenemissionscomputertomographie (FDG-PET/CT) innerhalb von 10 Wochen nach Studieneinschluss. Patienten aus der erwähnten gepoolten Analyse wurden ausgeschlossen. Die SBRT wurde im Falle peripherer Tumoren mit einer Gesamtdosis von 54 Gy in 3 Fraktionen oder im Fall zentraler Tumoren mit 50 Gy Gesamtdosis in 4 Fraktionen und einer simultanen lokalen Dosisaufsättigung bis 60 Gy auf das Tumorvolumen appliziert. Die Daten der operativ behandelten Patienten wurden prospektiv aus dem Kollektiv der Thorax- und Kardiochirurgie des Klinikums MD Anderson in Houston, Texas (USA), erhoben. Ausgewertet wurde das 3‑Jahres-Überleben als primärer Endpunkt mit einer Propensity-score-matching-Methode in einem Nichtunterlegenheitsdesign zwischen der SBRT-Gruppe und der operativ mittels VATS L-MLND therapierten Gruppe. Die Nichtunterlegenheit wurde festgestellt, wenn das 3‑Jahres-Überleben maximal um 12 % geringer war nach SBRT im Vergleich zu nach einer VATS L-MLND und die obere Grenze des 95 %-Konfidenzintervalls (KI) des Hazard Ratios (HR) geringer als 1,965 war. Das „propensity score matching“ basierte auf einem multivariablen logistischen Regressionsmodell mit den Kovariaten Alter, Tumorgröße, Histologie, Performance-Index, Interaktion von Alter und Geschlecht. Es wurde ein 5:1-digit-greedy-Zuordnungs-Algorithmus zur Paarfindung zwischen den beiden Gruppen verwendet. Weitere Endpunkte waren das 5‑Jahres-Überleben, das krebsspezifische Überleben, das progressionsfreie Überleben, die Rezidiv- und Fernmetastasierungsrate sowie die Toxizität.

## Ergebnisse

80 Patienten wurden eingeschlossen und deren Daten analysiert mit einer Nachbeobachtungszeit von 5,1 Jahren. Das Gesamtüberleben nach 3 Jahren belief sich jeweils auf 91 % (95 %-KI 85 %–98 %) nach SBRT und nach VATS L-MLND. Nach 5 Jahren betrug das Gesamtüberleben 87 % (79 %–95 %) nach SBRT und 84 % (76 %–93 %) nach VATS L-MLND. Es konnte kein signifikanter Unterschied des Gesamtüberlebens beobachtet werden (HR 0,86 [95 %-KI 0,45–1,65], *p* = 0,65).

Das progressionsfreie Überleben in beiden Gruppen nach 3 Jahren (80 %, 95 %-KI 72 %–89 % vs. 88 %, 81 %–96 %) und nach 5 Jahren (77 %, 68 %–87 % vs. 80 %, 71 %–90 %, Log-rank-*p* = 0,57) war ähnlich, ebenso wie das krebsspezifische Überleben.

Die Toxizität der SBRT war äußerst gering. Es wurden keine 4.- oder 5.-gradigen Nebenwirkungen beobachtet und jeweils nur ein Fall (1 %) von Grad 3 – Dyspnoe, Grad 2 – Pneumonitis und Grad 2 – Lungenfibrose detektiert. In der VATS-Gruppe waren die häufigsten Nebenwirkungen pulmonal (38 %) und kardiovaskulär (13 %). Eine postoperative intensivmedizinische Behandlung war nötig (1 %) und in 6 % erfolgte eine Rehospitalisierung.

In der VATS-L-MLND-Gruppe wurden in 10 % okkulte hiläre oder mediastinale Lymphknoten in der endgültigen postoperativen Pathologie festgestellt, welche im Staging zunächst nicht detektiert worden waren und im Anschluss eine adjuvante Therapie erhielten.

Die Lokalrezidivraten nach 5 Jahren unterschieden sich nicht zwischen den beiden Gruppen (6,3 % [95 %-KI 2,3 %–13,2 %] für SBRT vs. 1,3 % [0,1 %–6,2 %] für VATS, *p* = 0,1), ebenso nicht die Fernmetastasierungsraten (8,8 % [3,8 %–16,2 %] vs. 4,0 % [1,0 %–10,2 %], *p* = 0,19). Allerdings wurden häufiger regionale Rezidive in der SBRT-Gruppe beobachtet (12,5 % [95 %-KI 6,4 %–20,8 %] vs. 2,7 % [0,5 %–8,5 %], *p* = 0,017).

## Schlussfolgerung der Autoren

Die Autoren konnten feststellen, dass die SBRT der VATS L-MLND für operable NSCLC im Stadium IA nicht unterlegen ist (in Bezug auf das Gesamtüberleben), und empfehlen in diesen Fällen eine multidisziplinäre Therapieentscheidung.

## Kommentar

Die hier kommentierte Studie definiert den Stellenwert der SBRT für operable NSCLC in Stadium I, der bislang mangels randomisierter Studien auf Basis von großen Patientenkollektiven nur unzureichend belegt werden konnte.

Die SBRT bei Patienten mit NSCLC im Stadium I stellt mittlerweile einen radioonkologischen Therapiestandard dar und ist lange im klinischen Alltag erprobt. Insbesondere bei denjenigen Patienten, die nicht operiert werden können, ist die SBRT eine valide und gleichwertige Alternative. Nebst zahlreichen retrospektiven Arbeiten existieren allerdings nur wenige prospektive Studien (RTOG 0618 [1]; einarmig, nicht randomisiert, JCOG 0403 [Phase-II-Studie]) sowie die Daten aus der gepoolten Analyse der randomisierten STARS- und ROSEL-Studie [2].

Eine relevante Limitation bei der Erhebung von randomisierten Daten zum Vergleich der SBRT und der operativen Therapie stellt die mangelnde Rekrutierung aufgrund des Patienten- oder Behandlerwunschs dar (RTOG 1021, STARS: NCT00840749; ROSEL: NCT00687986, SABRTooth [3]). Nichtsdestotrotz konnten Chang et al. [2] in einer gepoolten Analyse der beiden unvollständig rekrutierten Studien ROSEL und STARS eine verbesserte Überlebensrate nach SBRT von 95 % vs. 79 % nach Lobektomie mit mediastinaler Lymphknotendissektion nach 3 Jahren ermitteln. Langzeitergebnisse lagen zu diesem Zeitpunkt noch nicht vor, das Patientenkollektiv war mit 58 Patienten klein, die beiden Studienprotokolle unterschieden sich und moderne Operationstechniken wie die VATS wurden nicht berücksichtigt.

In der hier kommentierten Studie versuchen nun die Autoren, diesen Limitationen zu begegnen durch eine erneute und erweiterte Erfassung der Patienten aus der überarbeiteten STARS-Studie und einem Propensity-score-matching-Vergleich mit einer prospektiv generierten Gruppe von Patienten, welche eine VATS L-MLND erhielten.

Die Ergebnisse des primären Endpunkts Gesamtüberleben sind mit 91 % nach 3 Jahren und je 87 % (SBRT) und 84 % (VATS L-MLND) nach 5 Jahren in beiden Gruppen nahezu identisch und exzellent. Ähnlich gut verhält es sich in beiden Gruppen mit dem progressionsfreien Überleben und dem krebsspezifischen Überleben.

Als bemerkenswert betrachten wir die Unterschiede in der therapiebezogenen Toxizität. In der SBRT-Gruppe war diese äußerst selten und 4.- und 5.-gradige Nebenwirkungen wurden überhaupt nicht beobachtet. In der Literatur werden tendenziell höhere Raten an pulmonalen Nebenwirkungen berichtet [4–7]. In einer aktuellen Übersichtsarbeit [7] werden die Raten an strahleninduzierten Lungentoxizitäten mit 10 % bis 15 % zusammengefasst. Diese hängen stark von dosimetrischen Faktoren wie der mittleren Lungendosis und dem Anteil der Lunge, welcher eine gewisse Mindestdosis erhält, als auch von klinischen Einflussgrößen wie einer interstitiellen Lungenerkrankung in der Patientenhistorie, der Tumorgröße, dem Stadium und der Tumorlokalisation ab. Allerdings überwiegen hier dennoch die Nebenwirkungen der operativen Therapie deutlich. Es wurden 38 % pulmonale und 13 % kardiovaskuläre Ereignisse registriert und außerdem 6 Rehospitalisierungen sowie 1 % postoperative intensivmedizinische Behandlungen. Diese Daten deuten also darauf hin, dass trotz modernster Operationsverfahren das Risiko von Nebenwirkungen im Vergleich zu einer SBRT erhöht ist. Diese Ergebnisse allein rechtfertigen unserer Meinung nach bereits jetzt den Einzug der hier diskutierten Studie in die multidisziplinären Tumorboards zur Therapieentscheidung sowie die Evaluation der Ergebnisse in prospektiven, randomisierten Studien, wobei jedoch die Realisierung weiterhin fraglich bleiben wird.

Einen diskutablen Aspekt stellt die 10 %ige Rate an positiven Lymphknoten in der VATS-L-MLND-Gruppe dar, welche erst in der postoperativen Pathologie auffiel, allerdings ohne Einfluss auf das Gesamtüberleben zu haben. Einerseits stellt dies die Qualität des Stagings infrage. Denn schließlich hat jeder Patient eine FDG-PET/CT erhalten und im Falle von Lymphknoten > 1 cm Durchmesser oder bei vermehrten PET/CT-Tracer-Uptake-Werten auch eine histologische Sicherung durch eine Feinnadelaspiration per endobronchialem Ultraschall. In der operativen Gruppe waren dies 60 % und in der SBRT-Gruppe 80 %. Die Rate an Regionalrezidiven war in der SBRT-Gruppe erhöht (12,5 % [95 %-KI 6,4 %–20,8 %] vs. 2,7 % [0,5 %–8,5 %], *p* = 0,017). Andererseits zeigt sich hier auch ein potenzieller Vorteil der operativen Behandlung, denn diese okkulten Lymphknoten blieben im Falle einer SBRT unentdeckt und konnten somit nicht einer speziellen Therapie zugeführt werden.

Es bleibt also zum einen festzuhalten, dass ein fundiertes N‑Staging therapierelevant ist. Zum anderen kommt die Frage nach der Sinnhaftigkeit einer prophylaktischen mediastinalen Lymphknotenbestrahlung unter kontroverser Abwägung der Vorteile und Risiken mit moderater Dosis und hochmoderner Präzisionstechnik auf. Dabei sei erneut an den fehlenden Einfluss auf das Gesamtüberleben in dieser Arbeit hingewiesen.

Ganz aktuell ist auch die rasante Zunahme der Behandlung mit systemischen, besonders immunologisch wirksamen Agenzien simultan oder sequenziell zur Radiotherapie. Allein für eine mögliche solche Folgebehandlung ist natürlich die Gewinnung einer Histologie sowohl im Falle einer geplanten SBRT als auch bei operativer Behandlung unentbehrlich.

## Fazit

Die Studie erhebt nicht den Anspruch, randomisierte, prospektive Daten ersetzen zu wollen. Auch stellt die primäre Beobachtung, also ein sehr gutes Gesamtüberleben in beiden Therapiearmen, keine neue Erkenntnis dar. Vielmehr bestätigt dies die Ergebnisse aus den vorherigen, teils statistisch unzureichenden oder retrospektiven Studien und wertet diese durch den sauber durchgeführten Vergleich mithilfe der Propensity-score-matching-Methode auf. Somit wird eine ziemlich solide Datenbasis generiert, die eine SBRT im Stadium I sehr stark unterstützt.

Die sekundären Endpunkte liefern hier den womöglich interessanteren und auch relevanteren Aspekt und werfen folgende Fragen auf: Welchen Stellenwert hat die diagnostische FDG-PET/CT für das Staging? Wie aussagekräftig ist die histologische Sicherung via Feinnadelaspiration und endobronchialem Ultraschall? In welchen Fällen ist eine prophylaktische mediastinale Lymphknotenbestrahlung sinnvoll? Welches Patientenkollektiv profitiert am ehesten von einer operativen Therapie in Anbetracht der hohen Nebenwirkungsraten?

Große randomisierte Studien wären natürlich wünschenswert, aber gerade in dieser Population ist eine Randomisierung oft nur schwer möglich. Daher können Arbeiten wie die hier vorliegende die Datenlage schön unterstützen und Therapieempfehlungen in diese Richtung legitimieren.

*Kim Melanie Kraus und Stephanie Elisabeth Combs, München*
